# Pro-oncogenic function of HIP-55/Drebrin-like (DBNL) through Ser269/Thr291-phospho-sensor motifs

**DOI:** 10.18632/oncotarget.1900

**Published:** 2014-04-16

**Authors:** Zijian Li, Hae Ryon Park, Zhi Shi, Zenggang Li, Cau Dinh Pham, Yuhong Du, Fadlo R. Khuri, Youyi Zhang, Qide Han, Haian Fu

**Affiliations:** ^1^ Department of Pharmacology, Emory University School of Medicine, Atlanta, GA, 30322, USA; ^2^ Institute of Vascular Medicine, Peking University Third Hospital, Key Laboratory of Cardiovascular Molecular Biology and Regulatory Peptides, Ministry of Health, Key Laboratory of Molecular Cardiovascular Sciences, Ministry of Education and Beijing Key Laboratory of Cardiovascular Receptors Research Beijing 100191, China; ^3^ Department of Hematology and Medical Oncology, Emory University School of Medicine and Winship Cancer Institute, Atlanta, GA, 30322, USA

**Keywords:** HIP-55, DBNL, HPK1, 14-3-3, tumorigenesis, signal transduction

## Abstract

HIP-55 (HPK1-interacting protein of 55 kDa, also named DBNL, SH3P7, and mAbp1) is a multidomain adaptor protein that is critical for organ development and the immune response. Here, we report the coupling of HIP-55 to cell growth control through its 14-3-3-binding phospho-Ser/Thr-sensor sites. Using affinity chromatography, we found HIP-55 formed a complex with 14-3-3 proteins, revealing a new node in phospho-Ser/Thr-mediated signaling networks. In addition, we demonstrated that HIP-55 is required for proper cell growth control. Enforced HIP-55 expression promoted proliferation, colony formation, migration, and invasion of lung cancer cells while silencing of HIP-55 reversed these effects. Importantly, HIP-55 was found to be upregulated in lung cancer cell lines and in tumor tissues of lung cancer patients. Upregulated HIP-55 was required to promote the growth of tumors in a xenograft animal model. However, tumors with S269A/T291A-mutated HIP-55, which ablates 14-3-3 binding, exhibited significantly reduced sizes, supporting a vital role of the HIP-55/14-3-3 protein interaction node in transmitting oncogenic signals. Mechanistically, HIP-55-mediated tumorigenesis activity appears to be in part mediated by antagonizing the tumor suppressor function of HPK1. Thus, the HIP-55–mediated oncogenic pathway, through S269/T291, may be exploited for the development of new therapeutic strategies.

## INTRODUCTION

The proper growth of mammalian cells is controlled by well-coordinated signal transduction pathways and networks. In addition to well-studied growth regulatory proteins with defined enzymatic activities, such as G proteins and kinases, the importance of adaptor proteins without catalytic functions in cell growth control has been increasingly recognized[1,2]. For example, the adaptor protein Grb2 contains an SH2 domain that binds phosphorylated Tyr and SH3 domains, which bind Pro-rich structures. In this configuration, Grb2 transmits signals to downstream effectors to promote cell proliferation upon growth factor stimulation. In addition, dimeric Grb2 can maintain an active, yet basal activity of FGFR2 prior to ligand engagement, suggesting another critical role of Grb2 in cell signaling [3]. In a similar manner, the family of 14-3-3 proteins can recognize phosphorylated Ser/Thr motifs to exert diverse regulatory functions [4-8]. Because of their vital role in controlling cell signaling, dysregulation of adaptor proteins, including 14-3-3, have been implicated in diverse diseases, including cancer. Thus, the identification of adaptor proteins that are associated with growth control may reveal new mechanisms that regulate cell proliferation under normal physiological conditions, as well as in cancer.

14-3-3 proteins are a family of adaptor proteins that control cell growth by binding to important regulatory proteins, including kinases, phosphatases, and other adaptor proteins [4,7,8]. In most cases, 14-3-3 binds client proteins that are phosphorylated at a specific Ser or Thr motif, such as RSxpS/TxP. Because of their important roles in cell growth control, dysregulation of 14-3-3 proteins has been associated with tumorigenesis. Proteomics studies have been carried out to understand how 14-3-3 interactions regulate diverse cellular processes [9-11]. Such studies have revealed a large number of 14-3-3-interacting client proteins, which may be important to transmit 14-3-3-mediated growth regulatory signals and oncogenic activities. Thus, functional characterization of newly identified 14-3-3–associated regulatory proteins may not only offer critical insights into how 14-3-3 serves as a master regulator of signal transduction, but also may define the role of less-studied proteins in cell growth regulatory networks for mechanistic and therapeutic discoveries.

Here, we report the functional characterization of hematopoietic progenitor kinase 1 (HPK1)-interacting protein of 55 kDa (HIP-55)/Drebrin-like (DBNL), which was identified in a 14-3-3γ affinity resin-based proteomic approach using lung adenocarcinoma A549 cells. HIP-55 is a multi-domain adaptor protein with an established role in endocytosis and immune responses[12-14]. By manipulating the levels of HIP-55 in cells and in a xenograft animal system, our studies reveal a role of HIP-55 in cell growth control, and suggest the potential importance of dysregulated HIP-55 in tumorigenesis and tumor progression through a mechanism involving the 14-3-3 binding phospho-sensor sites, S269 and T291.

## RESULTS

### HIP-55 binds to 14-3-3 proteins

**Figure 1:**
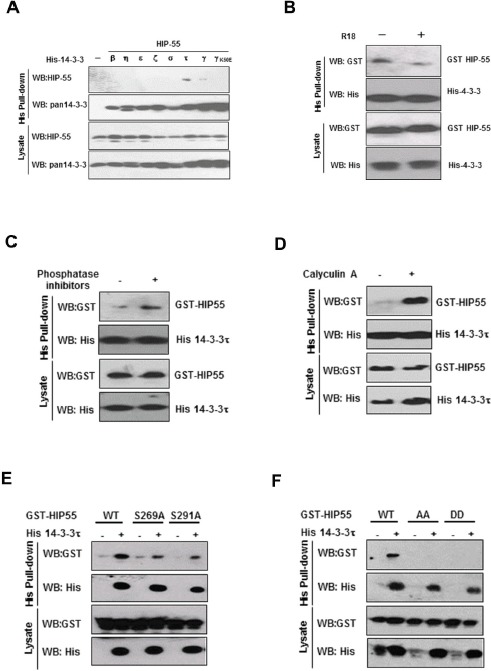
HIP-55 interacts with 14-3-3 through S269 and T291 (A) Interaction of HIP-55 with isoforms of 14-3-3 proteins. HEK293 cells were co-transfected with each of the seven different 14-3-3 isoforms and HIP-55, lysed after 48 h, and subjected to His pull-down assays. Bound proteins were examined by Western blot analysis. (B) The HIP-55/14-3-3 association is mediated by the amphipathic groove of 14-3-3 (R18 competition). HEK293 cells were co-transfected with HIP-55 and 14-3-3τ. After 36 h, the cells were harvested and incubated for 30 min at 4°C with DMSO alone (−) or R18 (+). Cell lysates were subjected to His pull-down, followed by Western blot analysis with the indicated antibodies. (C) The presence of general phosphatase inhibitors preserves the HIP-55/14-3-3 association. Lysates from HEK293 cells co-expressing His-14-3-3τ and HIP-55 were incubated with or without phosphatase inhibitors for 0.5 h at room temperature, followed by Western blot analysis to detect GST-HIP-55 in the His-14-3-3τ complexes. (D) Inhibition of Ser/Thr phosphatases, PP1 and PP2A, by Calyculin A enhances HIP-55/14-3-3 binding. HEK293 cell lysates co-expressing His-14-3-3τ and HIP-55 were incubated with or without Calyculin A for 0.5 h at room temperature, followed by Western blot analysis with the indicated antibodies. (E) Ser269 and Thr291 of HIP-55 are required for 14-3-3 binding. HEK293 cells were co-transfected with His-14-3-3τ and the indicated GST-HIP-55 vectors. His-14-3-3τ complexes were pulled-down from cell lysates and GST-HIP-55 variants were detected by Western blot. Mutation of either S269 or T291 of HIP-55 decreased 14-3-3 binding. (F) Double mutants S269/T291AA and DD of HIP-55 completely prevent the binding of 14-3-3.

To identify 14-3-3 binding proteins that are associated with tumorigenesis, we utilized A549 lung cancer cells as a protein source for a 14-3-3γ affinity column-based proteomic strategy. The γ isoform of 14-3-3 proteins was initially chosen due to its demonstrated role in cell growth control[15]. Briefly, A549 cell lysates were incubated with GST-14-3-3γ affinity resin. After washing, the well-characterized 14-3-3 antagonist peptide, R18, was used as a competitive reagent to elute specific 14-3-3 binding proteins. Analysis of the eluted proteins by mass spectrometry indicated that one of the associated proteins was HIP-55[12-14]. To confirm this result, an alternative hexa-histidine-14-3-3-based pull-down assay was employed. As there are seven known 14-3-3 isoforms, we included the remaining six isoforms to expand our observations beyond 14-3-3γ. As expected, HIP-55 was specifically associated with 14-3-3γ while failing to bind to 14-3-3γ/K50E, a known mutant with reduced ligand binding activity [16](Fig. 1A). Among the seven isoforms, 14-3-3τ showed the strongest interaction with HIP-55. These results demonstrate that HIP-55 has an isoform preference towards14-3-3τ, which was selected for the subsequent studies. R18 effectively inhibited the HIP-55 interaction with 14-3-3 τ (Figure 1B), further substantiating the binding specificity[17,18]. The competition between R18 and 14-3-3τ for HIP-55 interaction supports the binding of HIP-55 to the defined amphipathic groove in 14-3-3τ

### HIP-55 S269 and T291 are required for its association with 14-3-3

14-3-3 typically binds to proteins containing defined phospho-Ser or phospho-Thr motifs[7, 8]. To determine whether the interaction of HIP-55 with 14-3-3 is phosphorylation-dependent, we performed 14-3-3/HIP-55 binding assays in the presence or absence of general phosphatase inhibitors [40]. HIP-55 binding to 14-3-3 was dramatically diminished in the absence of phosphatase inhibitors (Figure 1C), indicating that global inhibition of phosphatases maintains the HIP-55/14-3-3 interaction. In particular, the HIP-55/14-3-3 interaction was enhanced by calyculin A, an inhibitor of the PP1 and PP2A phosphatases (Figure 1D). Taken together, these results indicate that HIP-55 interacts with 14-3-3 in a phosphorylation-dependent manner.

To determine the site(s) in HIP-55 that mediates 14-3-3 binding, we searched for conserved sequences in HIP-55 by using the Scansite Motif Scanner (http://scansite.mit.edu/) and identified several potential 14-3-3 binding sites including S269 and T291. To experimentally validate the 14-3-3 binding sites in HIP-55, we mutated these Ser/Thr sites to Ala and examined the binding of the HIP-55 mutants to 14-3-3 using a hexa-His-14-3-3 pull-down assay. Compared with HIP-55 WT, the HIP-55 mutants, S269A and T291A, showed reduced interaction with 14-3-3 (Figure 1E), while other mutations had no significant effect (Data not shown). These results indicate that both the S269 and T291 sites are involved in the binding of HIP-55 to 14-3-3. Indeed, mutation of both sites, either HIP-55/S269A/T291A (AA) or HIP-55/S269D/T291D (DD), abolished the binding of HIP-55 to 14-3-3 (Figure 1F). These results indicate that HIP-55 binds 14-3-3 through both the S269 and T291 sites.

### HIP-55 promotes cancer cell survival and anchorage-independent growth

**Figure 2:**
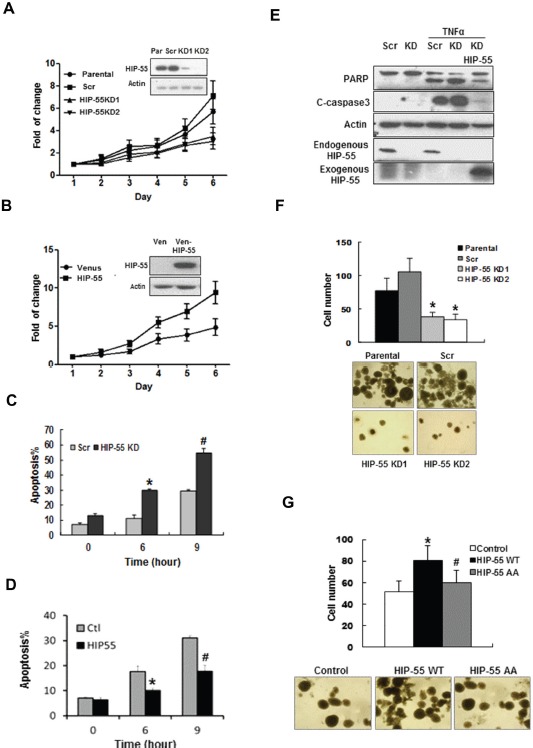
HIP-55 promotes cancer cell survival (A) Knockdown of HIP-55 decreases lung cancer cell growth. Cells were infected with retroviruses carrying either scrambled control shRNA (Scr) or HIP-55 shRNA (KD1 or KD2) as indicated. Cell growth assays were performed using sulforhodamine-B (SRB). (B) Overexpression of HIP-55 increases cell growth. Cells were infected with retroviruses carrying either a control Venus expression vector (Ven) or Venus-HIP-55 expression vector as indicated. Cell growth assays were performed using SRB. (C) Silencing of HIP-55 increases cell apoptosis. HIP-55 knockdown(KD) cells and scrambled (Scr) cells were treated with TNFα/CHX for the indicated times. Then cells were collected and stained with 7-AAD and Annexin V-PE, and analyzed by flow cytometry. *P<0.05 HIP-55KD vs. Scr (6 hours); #P<0.05 HIP-55KD vs. Scr (9 hours). (D) Overexpression of HIP-55 attenuates apoptosis. HIP-55 overexpression cells and control cells were treated with TNFα/CHX for the indicated time. Live cells were collected, stained with Annexin V and 7-AAD, and subjected to flow cytometry. *P<0.05 HIP-55 vs. Ctl (6 hours); #P<0.05 HIP-55 vs. Ctl (9 hours). (E) HIP-55 levels impact apoptosis readouts. HIP-55 knockdown or overexpressing cells were treated with TNFα/CHX for 9 h and harvested for Western blotting with antibodies specific for PARP or cleaved caspase 3 (C-caspase 3). (F) Silencing of HIP-55 decreases the colony formation activity of lung cancer cells. Cells on soft agar plates were grown for 3 weeks before colonies were stained and visualized microscopically. A representative view of each cell line is shown (lower panel). Quantification of the colony formation data is shown in the upper panel. *P<0.05 HIP-55KD vs. Scr. (G) HIP-55 overexpression increases the colony formation activity of lung cancer cells. Cells on soft agar plates were grown for 3 weeks before colonies were stained and visualized microscopically. A representative view of each cell line is shown (lower panel). Quantification of the colony formation data is shown in the upper panel. *P<0.05 HIP-55WT vs. Control; #P<0.05 HIP-55 AA vs. HIP-55WT.

14-3-3 proteins often regulate cell growth through controlling the pro-survival function of their client proteins[19]. It is possible that HIP-55 mediates a 14-3-3–controlled growth regulatory mechanism. Therefore, we investigated the requirement of HIP-55 for lung cancer cell growth. For this purpose, HIP-55 was knocked down in A549 lung adenocarcinoma cells via a retroviral-based shRNA expression system, and the effect of downregulating HIP-55 protein on the proliferation and apoptosis potential of these cells was monitored. Knockdown of HIP-55 resulted in a significant decrease in cell proliferation, suggesting a role of HIP-55 in maintaining cell viability (Figure 2A). Consistent with this finding, overexpression of HIP-55 significantly increased the growth rate of A549 cells (Figure 2B).

It is possible that HIP-55 supports cell growth in part by antagonizing apoptotic signaling [19]. Thus, we examined the effect of HIP-55 on the levels of apoptosis induced by well-established extrinsic death signals, tumor necrosis factor (TNF)α/cycloheximide (CHX)[20]. While TNFα/CHX treatment induced apoptotic cell death in a time-dependent manner as previously reported, cells with shRNA-silenced HIP-55 showed significantly increased levels of apoptosis as revealed by Annexin V staining (Figure 2C). Conversely, overexpression of HIP-55 resulted in a significantly reduced TNFα/CHX-induced apoptotic response (Figure 2D). Moreover, results from a caspase-3 activation assay indicated an enhanced apoptotic effect in cells with HIP-55 knockdown (Figure 2E). Importantly, this effect could be reversed by co-expression of exogenous HIP-55, as demonstrated by reduced PARP cleavage and a decrease in cleaved, active caspase 3 (Figure 2E). These results suggest a critical role of HIP-55 in supporting cell survival and antagonizing apoptosis.

Tumor cells often gain anchorage-independent survival and growth capabilities[21]. These properties of cancer cells can be examined by the ability to form colonies in a semi-solid medium [22]. Using a soft agar colony formation assay, we found that knockdown of HIP-55 with specific shRNAs dramatically reduced both the number and size of the colonies formed as compared to parental cells or cells with scrambled shRNA control (Figure 2F). Conversely, overexpression of HIP-55 reversed this effect (Figure 2G). Taken together, these results demonstrate that HIP55 promotes cell growth and enhances the anchorage-independent survival of lung cancer cells, and support a role for HIP-55 in promoting tumorigenesis.

### HIP-55 is required for cancer cell migration and invasion

**Figure 3:**
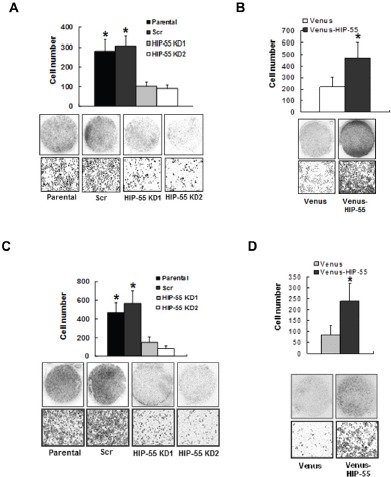
HIP-55 promotes cancer cell migration and invasion Effect of HIP-55 knockdown (A) and HIP-55 overexpression. (B) on A549 cell migration. Cells that migrated through the transwell were fixed, stained with Hema-3, and images captured using a scanner (upper panel of images) or phase-contrast microscopy (×10 magnification, lower panel of images). Effect of HIP-55 knockdown (C) and HIP-55 overexpression (D) on A549 cell invasion. The cells that migrated through the Matrigel-coated chambers were fixed, stained with Hema-3, and images captured using a scanner (upper panel of images) or phase-contrast microscopy (×10 magnification, lower panel of images). *P<0.05.

To assess the importance of HIP-55 in cell migration, as required for tumor progression, we used transwell. The transwell assay demonstrated that HIP-55 knockdown led to decreased cell migration (Figure 3A). On the other hand, overexpression of HIP-55 resulted in an increase in cell migration (Figure 3B). Consistent with the results from the transwell assay, cell motility following wound generation in a scratch wound-healing assay was reduced by shRNA-based knockdown of HIP-55. Closure of the scratched wound was almost complete in parental and control cells with scrambled shRNA at the 72 h time point, while the scratched wound gap remained unchanged in HIP-55 knockdown cells (Data not shown). These results indicate that HIP-55 can promote lung cancer cell migration. Increased migration promoted by HIP-55 may enable the invasion of cancer cells through the basement membrane, a key event during metastasis. Thus, we evaluated the effect of HIP-55 on cell invasion ability. As shown in Figure 3C, the silencing of HIP-55 expression significantly reduced the invasive potential of A549 cells. In support of its role in cell invasion, overexpression of HIP-55 greatly increased the invasive potential of A549 cells as compared to vector control cells (Figure 3D). Thus, our results indicate that HIP-55 is required for the acquisition and maintenance of multiple properties that are important for tumorigenesis and progression.

### HIP-55 promotes tumor formation *in vivo* in a S269/T291-dependent manner

**Figure 4:**
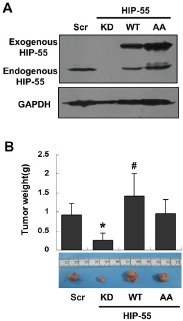
HIP-55 promotes tumor formation *in vivo* in a S269/T291-dependent manner (A) A549 cells were infected with lentiviruses carrying scrambled control, HIP-55-specific shRNA, HIP-55WT cDNA, or HIP-55AA cDNA, and (B) were subcutaneously inoculated into 5-week-old female SCID mice (n = 6). Seven weeks later the mice were sacrificed and the tumors were weighed and analyzed. *P<0.05 HIP-55KD vs. Scr; #P<0.05 HIP-55 WT vs. HIP-55AA

To determine the effect of HIP-55 on tumor growth *in vivo*, we used a xenograft animal model with either reduced or enhanced expression of HIP-55 protein. To examine the requirement for HIP-55 in tumorigenesis, mice were injected with A549 lung cancer cells expressing control scrambled shRNA or HIP-55-specific shRNA. While A549 cells expressing control shRNA induced tumor formation, knockdown of HIP-55 decreased the tumorigenic activity of A549 cells resulting in dramatically reduced tumor volume (Figure 4A and 4B). Next, the effect of upregulated HIP-55 on tumor formation was examined by injecting mice with A549 cells overexpressing WT HIP-55. As shown in Figure 4A and 4B significant increases in tumor size and weight were observed in animals with HIP-55 overexpression, indicating that HIP-55 overexpression enhances tumor growth in SCID mice. Finally, to examine the importance of the HIP-55/14-3-3 interaction in tumorigenic functions, A549 cells expressing the HIP-55 AA mutant (S269A/T291A) were injected into mice. In contrast to WT HIP-55, the HIP-55/AA mutant exhibited reduced promotion of tumor growth in SCID mice which is consistent with results from the in vitro colony formation assay as shown in Figure 2G, demonstrating that HIP-55 promotes tumor growth *in vivo* likely through a HIP-55/14-3-3 complex-dependent mechanism.

### HIP-55 antagonizes HPK1–dependent functions

**Figure 5:**
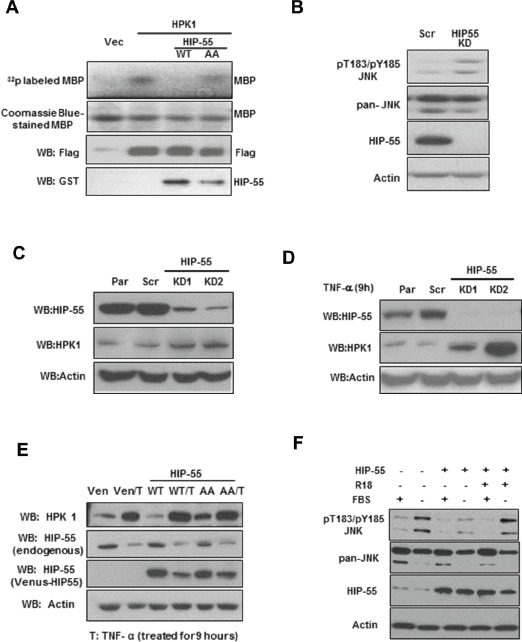
HIP-55/14-3-3 negatively regulates HPK1 kinase activity (A) HIP-55 inhibits HPK1 kinase activity in a S269/T291-dependent manner. HEK293 cells were co-transfected with the indicated vectors for 36 h, at which time the cells were lysed and a Flag IP was performed to isolate HPK1 kinase. Isolated protein complexes were incubated in an *in vitro* kinase assay using MBP as a HPK1 substrate. The amount of HPK1 used in each assay was validated by Western blot. (B) Knockdown of HIP-55 is correlated with increased HPK1-JNK signaling in lung cancer cells. HIP-55 knockdown A549 cells and scrambled control-expressing A549 cells were harvested for Western blot analysis of JNK. (C) Knockdown of HIP-55 is correlated with increased HPK1 level. A549 cells with different levels of HIP-55 (parental, shRNA scr or HIP-55 knockdown) were lysed. The expression level of HIP-55, as well as HPK1 and actin levels, were examined by Western blotting with specific antibodies. (D) TNFα exacerbates the HIP-55 knockdown effect in stabilizing HPK1. Similar experiments as in (C) were carried out in the presence of TNFα. (E) HIP-55 overexpression promotes HPK1 degradation in a S269/T291-dependent manner. Cells overexpressing control Venus, Venus-HIP 55WT or Venus-HIP 55AA cells were harvested, HPK1 and HIP-55 expression were analyzed by Western blotting. The overexpression of HIP-55 WT, but not the 14-3-3 binding-defective HIP-55(HIP-55AA) mutant, promotes HPK1 degradation. Cells treated with TNFα reduced HIP-55, countered HIP-55 effect and increased the expression of HPK1. (F) The 14-3-3 antagonist peptide, R18, reversed HIP-55 inhibition of pT183/pY185JNK. Cells incubated in FBS-free medium exhibited JNK phosphorylation that was inhibited by HIP-55 overexpression. Treatment with a 14-3-3 antagonist peptide reversed the HIP-55-mediated effect on JNK phosphorylation.

To begin to address the mechanism by which HIP-55 contributes to tumorigenesis, a potential role of HIP-55 in regulating its effector HPK1-mediated function was examined [12,23]. HPK1 inhibits cell proliferation and induces apoptosis with a proposed tumor suppressor function [24,25]. Thus, it is possible that HIP-55 interacts with HPK1 and antagonizes its growth suppressive activity in tumors. To test this model, a direct effect of HIP-55 on the catalytic activity of HPK1 was investigated using an in vitro radiolabeling kinase assay. FLAG-tagged HPK1 was immunoprecipitated from cells with co-expressed HIP-55 and used for the kinase assay. The HPK1 kinase assay was performed using myelin basic protein (MBP) as a substrate and monitoring HPK1-catalyzed γ-*^32^*P transfer from *^32^*P-ATP to generate 32P-MBP. As shown in Fig. 5A, HIP-55 co-expression resulted in a decrease in the kinase activity of HPK1, suggesting a negative regulatory effect of HIP-55 on HPK1. Interestingly, co-expression of the S269A/T291A HIP-55 AA mutant showed little inhibitory effect on HPK1 kinase activity (Fig. 5A), suggesting the importance of S269/T291 phosphorylation in the inhibition of HPK1's catalytic activity. To confirm the inhibitory effect of HIP-55 on HPK1 in cells, the effect of knockdown of HIP-55 on a HPK1 effector protein, JNK, was monitored[24,25]. Indeed, cells with knockdown of HIP-55 exhibited increased phosphorylation of the physiological HPK1 substrate, JNK (Figure 5B).

Through Western blot analysis, we observed that HIP-55 and HPK1 levels were inversely correlated. To test whether HIP-55 regulates HPK1 protein level, in addition to HPK1 catalytic activity, we utilized two approaches to manipulate HIP-55 level, (i) an shRNA silencing approach and (ii) a HIP-55 negative regulatory signaling molecule, TNFα. TNFα offers an alternative approach to reduce HIP-55 without exogenous manipulation with shRNA. As shown in Figure 5C, when HIP-55 protein was reduced by shRNA, the expression of HPK1 increased. This effect was exacerbated when TNFα was applied to cells to further downregulate HIP-55 protein (Figure 5D). Conversely, overexpression of HIP-55 was correlated with drastically reduced HPK1 (Figure 5E). The HIP-55 effect appears to depend on the integrity of S269 and T291, as the HIP-55 AA mutant S269A/T291A reduced HPK-1 levels to a lesser extent (Figure 5E). TNFα, which can reduce HIP-55 protein levels, counters the HIP-55 overexpression effect and maintains HPK1 stability. To further confirm that HIP-55-mediated suppression of HPK1 activity depends on binding to 14-3-3, a 14-3-3 antagonist peptide, R18, was used. As a functional readout of HPK1 activation, the phosphorylation state of JNK at T183/Y185 was monitored in cells treated with various agents. As expected, serum (FBS) withdrawal induced upregulation of pT183/pY185 JNK, which was attenuated by HIP-55 overexpression (Figure 5F). This HIP-55-induced pJNK inhibition was reversed by the expression of R18. These results support the involvement of 14-3-3 proteins in the regulation of HPK1-JNK signaling by HIP-55. Taken together, these experiments suggest that HIP-55 may promote tumorigenesis in part through negative regulation of the HPK1 pathway.

### HIP-55 is upregulated in cancer cells and tumors from lung cancer patients

**Figure 6:**
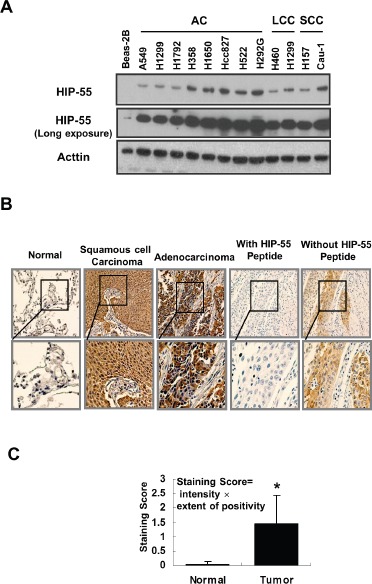
HIP-55 is upregulated in lung cancer cells and specimens lung cancer patients (A) HIP-55 expression in lung cancer cell lines. Cell lysates were prepared from the indicated lung cancer cell line or control BEAS-2B cells and separated by SDS/PAGE. HIP-55 expression was examined by Western blotting with a HIP-55-specific antibody. (B) Expression of HIP-55 in lung cancer tissue and normal lung tissue samples. Immunohistochemistry was performed on slides from paired fixed tissue samples using an anti-HIP-55 antibody (×10 magnification.). Incubation with a HIP-55 peptide antigen blocked HIP-55 staining of patient samples. (C) Quantification of HIP-55 expression in tumor samples. Lung tumor and normal tissue samples were analyzed by immunohistochemistry using specific anti-HIP-55 antibodies and scored in a blinded fashion.

To determine the pathophysiological relevance of HIP-55 in cancer, we examined whether HIP-55 expression is dysregulated in human cancers. The expression of HIP-55 protein was probed in a panel of lung cancer cell lines including those from adenocarcinomas (AC), large cell carcinomas (LCC), and squamous cell cancers (SCC), as well as the cultured human normal bronchial epithelial cell line, BEAS-2B. As shown in Figure 6A, HIP-55 protein expression levels were higher in lung cancer cells than in normal control cells, suggesting that HIP-55 is dysregulated in tumors. To address whether HIP-55 is dysregulated in patient tumors, we examined the expression levels of HIP-55 in lung tumor tissues in comparison to normal lung tissues using immunohistochemical staining (IHC). For this purpose, the specificity of the HIP-55 antibody is essential. To confirm the specificity of the HIP-55 antibody, a peptide antigen from HIP-55 was included in our studies and was shown to completely block the IHC staining signal (Figure 6B). To quantify HIP-55 expression in tumors, both staining intensity and percent stained area were used to score for positives. Statistical analysis of the scores for the specimens demonstrated that HIP-55 expression was significantly increased in tumor tissues as compared to normal lung specimens (Figure 6C). This upregulation of HIP-55 may contribute to the acquisition of tumorigenic properties in lung cancer.

## DISCUSSION

Using 14-3-3γ–based affinity chromatography, we identified HIP-55 as a 14-3-3-binding protein with preference for the τ isoform. In further support of this interaction, the HIP-55/14-3-3 association was found to be phosphorylation-dependent. Through mutagenesis studies, we mapped the 14-3-3 binding sites to S269 and T291 within motif 1 (RAMS269TTS) and motif 2 (KQLT291QP), respectively. Through S269 and T291 phosphorylation and 14-3-3 binding, HIP-55 may serve as a signal sensor and transmitter to regulate kinase-mediated signaling networks. Thus, the S269 and T291 motifs offer novel regulatory elements through which HIP-55 serves as a multi-functional signaling hub, along with its known actin-binding domain and poly-proline binding SH3 domain [12-14, 26]. The work described here provides an entirely independent validation of the reported HIP-55/14-3-3 interaction using a lung cancer experimental system, which represents a disease with limited therapeutic options [27,28]. Importantly, our functional studies support a critical role for HIP-55, a signaling adaptor protein, in promoting the growth of lung cancer cells and in acquiring tumorigenic properties, including anchorage-independent colony formation and tumor formation in a xenograft animal model.

Through a series of loss-of-function (shRNA silencing) and gain-of-function (overexpression) experiments, we established that HIP-55 is required to maintain cell survival. While downregulation of HIP-55 is associated with a reduced growth rate and an increased apoptotic potential of lung cancer cells, restoring HIP-55 expression reverses the growth regulatory effect. These results are supported by reported functions of HIP-55. As a key component of the immunological synapse, HIP-55 regulates T-cell activation and proliferation[29]. HIP-55-−/− mice were viable and fertile, but showed decreased body weight and increased occurrence of death within the first 4 weeks after birth, and further suffered from splenomegaly, heart failure, lung edema, and behavioral abnormalities [29].

The demonstrated pro-growth activity of HIP-55 implies its potential role in neoplastic development. Indeed, HIP-55 is overexpressed in a large number of lung cancer cells and in tumor tissues derived from lung cancer patients. Persistent expression of HIP-55 appears to be required for the acquisition of tumor-associated properties such as anchorage-independent colony formation. In support of its involvement in tumor progression, knockdown of HIP-55 attenuated the migration and invasion abilities of lung cancer cells. A role of HIP-55 in promoting tumor progression is also supported by observations in other cancers. For example, HIP-55 is highly expressed in samples from squamous cervical cancer patients and those with pancreatic cancer with lymph node metastasis [30,31]. In our study, the level of HIP-55 was correlated with the tumor growth of A549 cells in a xenograft animal model, suggesting a central role for this adaptor protein in supporting lung cancer development in mice. Importantly, this tumor promoting activity was associated with the integrity of the 14-3-3 binding S269 and T291 phospho-sensor sites. Mutating these sites (S269A/T291A) reduced the ability of HIP-55 to enhance tumor growth (Fig. 4). These data suggest a critical linkage of the S269 and T291 phosphorylation sites with the pro-oncogenic activity of HIIP-55, likely through inducing 14-3-3 protein complex formation. It is possible that the S269 and T291 sites sense the activity of an oncogenic kinase(s) to regulate 14-3-3 interaction and couple this molecular event to the tumorigenic machinery for the control of tumorigenesis and tumor progression[32,33].

One possible mechanism by which HIP-55 regulates tumorigenesis is through its interaction with HPK1[12]. HPK1 is a MAP4K that activates JNK and plays an important role in regulating stress response, proliferation, and apoptosis in hematopoietic cells [23-25]. In pancreatic cancer, HPK1 appears to have a tumor suppressor function possibly through increased p21 and p27 [34]. Loss of HPK1, through proteasome-mediated degradation, may lead to enhanced tumorigenesis and an increase in the invasive phenotype of pancreatic cancer. In lung cancer, HIP-55 overexpression appears to be correlated with reduced HPK1 levels, which may lead to diminished HPK1 tumor suppressor activity. Indeed, HIP-55 levels are inversely correlated with HPK1 in lung cancer cells (Fig. 5). In addition, HIP-55 also has a direct mechanism to constrain the catalytic activity of HPK1, ensuring a robust inhibitory function against HPK1. It is interesting to note that it is generally difficult to target a tumor suppressor for therapeutic development, as inhibiting oncogenic activity is usually simpler than enhancing a tumor suppressor using a small molecule compound[35]. The interaction of tumorigenic HIP-55 with tumor suppressive HPK1 may offer a novel strategy to target HIP-55-mediated cancers. For example, inhibition of HIP-55 or disruption of the HIP-55/HPK1 interaction may lead to restoration of the tumor suppressor activity of HPK1 and exert potential therapeutic benefits.

The identification of HIP-55 as a pro-oncogenic protein reveals a potential new target for therapeutic discovery. The demonstrated importance of the S269/T291-phosphosensor sites of HIP-55 in mediating 14-3-3 binding and acquired tumorigenic properties of lung cancer cells supports HIP-55 as a novel oncogenic signaling hub that integrates multiple growth regulatory cues through defined functional domains, actin-binding, phospho-sensing, and SH3 modules[23,36-40]. Delineating how HIP-55 is regulated under physiological conditions and dysregulated in tumors through these signaling modules may reveal tumor-specific strategies to develop HIP-55-targeted therapeutic agents for the treatment of cancer patients.

## MATERIALS AND METHODS

### Plasmids and reagents

HIP-55 was amplified by PCR and cloned into Gateway expression vectors to generate epitope-tagged HIP-55 proteins (Life Technologies)[41]. Anti-HIP-55 (#612614) antibody was purchased from BD Bioscience (San Diego, CA). Anti-HPK1 (H-200), anti-GST (Z-5), anti-HA (F-7), Anti-His (H-15), and anti-14-3-3 (K-19) antibodies were from Santa Cruz Biotechnology (Santa Cruz, CA). Anti- Phospho-JNK(pT183/Y185; #4668) and Anti-JNK (#9258) were from Cell Signaling Technologies (Beverly, MA).

### Cell culture and transfection

The human A549 non-small cell lung cancer (NSCLC) cell and HEK293 cells were maintained in DMEM with 10% fetal bovine serum and 100 units penicillin-streptomycin at 37°C with 5% CO2 in a humidified incubator. For transient expression, HEK293 cells were transfected with recombinant DNA plasmids using Fugene HD (Roche) following the manufacturer's protocol. Unless otherwise indicated, cells were harvested two days post-transfection for the pull-down assay and coimmunoprecipitation assay.

### Generation of HIP-55 knockdown (KD) stable cell lines

Recombinant retroviruses carrying HIP-55 shRNA were prepared with pSilencer 5.1 (Ambion). Two distinct sequences for HIP-55 were selected: AATGGCCTGGTGATTCCCACA and AACAGTGAACGTAGAGAATTG. To generate HIP-55-silenced stable cell lines, infected cells were subjected to selection in the presence of puromycin (1.25 µg/ml). Drug-resistant clones were collected, pooled, and expanded. HIP-55 expression level was verified by Western blot analysis for each experiment.

### Site-directed mutagenesis

A QuikChange™ site-directed mutagenesis kit (Stratagene) was used to generate mutations in HIP55 following the manufacturer's protocol. Two complementary mutagenic primers were used for each mutation. The forward mutagenic primers used were: S269A: 5'-GAAGGAGAGGGCCATGG

CCACCACCTC-3'; T291A: 5'-CCTG CAGAAGCAGCTAGCCCAACCAGAGACCC-3'.

### Affinity pull-down assays

Hexa-His pull-down: Cells were lysed 48 hours post-transfection in His pull-down lysis buffer (1% Nonidet P-40, 137 mM NaCl, 1 mM MgCl2, 40 mM Tris-Cl, 60 mM imidazole, 5 mM Na4P2O7, 5 mM NaF, 2 mM Na3VO4, 1 mM phenylmethylsulfonyl fluoride, 10 mg/L aprotinin, 10 mg/L leupeptin) [42,43]. Lysates were cleared by centrifugation at 4°C. The clarified cell lysate was incubated with nickel-charged resin for 2 hours at 4°C. The resin was washed 2 times with washing buffer (500 mM NaCl, 20 mM Tris-Cl, 60 mM imidazole) and once with binding buffer (500 mM NaCl, 20 mM Tris-Cl, 5 mM imidazole). Bound proteins were released from the resin by boiling in 6X SDS-PAGE sample buffer.

GST pull-down: Cells were lysed in GST pull-down lysis buffer (1% Nonidet P-40, 150 mM NaCl, 100 mM Hepes, 5 mM Na4P2O7, 5 mM NaF, 2 mM Na3VO4, 1 mM phenylmethylsulfonyl fluoride, 10 mg/L aprotinin, 10 mg/L leupeptin). Cleared cell lysates were incubated with glutathione-conjugated Sepharose for 2 hours at 4°C. The resin was then washed 3 times with GST pull-down lysis buffer and boiled in 6X SDS-PAGE sample buffer.

### Co-immunoprecipitation and Western blotting

Whole-cell extracts were prepared in immunoprecipitation buffer (1% Nonidet P-40, 150 mM NaCl, 100 mM Hepes, 5 mM Na4P2O7, 5 mM NaF, 2 mM Na3VO4, 1 mM phenylmethylsulfonyl fluoride, 10 mg/L aprotinin, 10 mg/L leupeptin) and immunoprecipitated with or without the appropriate antibody and protein G conjugated Sepharose for 2 hours at 4°C [41]. Precipitated proteins were washed three times in immunoprecipitation buffer, loaded onto 12.5% Tris–glycine gels, and transferred for 3h onto immunoblot polyvinylidene difluoride (PVDF) membranes (Bio-Rad, Hercules, CA). Proteins were detected using West-Pico or West-Dura enhanced chemiluminescent detection reagents (Pierce) and a Kodak imager or film-based system.

### HPK1 kinase assay

To examine HPK1 activity, HPK1, isolated from lysates through immunoprecipitation, was incubated with 10 µCi of [γ-32P]ATP and 5 µg of myelin basic protein (MBP) in 25 µl of kinase buffer (50 mM HEPES, 5 mM MgCl2, 10 mM dithiothreitol) [17]. All reactions were incubated at 30°C for 15 min and terminated by addition of sample buffer. Proteins were separated by 10% SDS-PAGE, and phosphorylation was visualized by autoradiography.

### Soft agar colony formation assay

Cells (1× 103) were resuspended in RPMI 1640 medium (1.0 ml with 20% FBS and 0.33% agar) and plated over a layer of solidified RPMI 1640 medium/20% FBS/0.66% agar (2.0 ml) [19]. Plates were incubated at 37°C, and colonies were scored in a blinded fashion.

### Apoptosis and cell viability assays

For analysis of apoptosis, cells were harvested, washed with PBS, and then stained with 7-AAD and Annexin V-PE. Samples were analyzed using a Guava flow cytometry system (Millipore). Cell viability is monitored with quantifying cellular proteins by the sulforhodamine B (SRB) assay. Cells were fixed with ice cold trichloroacetic acid (10% (wlv) and incubated for 60 min at 4°C. After washing with deionized water and air dried, SRB (Sigma) solution (0.4% w/v in 1% acetic acid) was added and incubated for 10 min at room temperature. After removing unbound SRB and washing with 1% acetic acid, cells were air dried. Bound protein stain was solubilized with of 10 mM Tris base (pH 10.5; 100 μ1), and OD565 nm was recorded using a microtiter plate reader.

### Invasion and migration assays

For invasion assays, cell-culture inserts (8µm pore size; BD Biosciences) were evenly coated with diluted matrigel. Cells were placed in serum-free medium for 24 h before experiments. Cells (1×105) were layered in the upper compartment of transwell inserts. Medium containing 10% FBS was used in the bottom wells. After 48 hours at 37°C in a 5% CO2 incubator, the matrigel coating on the upper surface of the filter was wiped off using a cotton swab. Cells that migrated through the filters were fixed, stained with Hema-3, photographed, and the number of cells that invaded was counted for quantification. The results represent the average and standard deviation of three independent experiments. The cell migration assay was similar to the invasion assay except that the inserts were not coated with matrigel. For cell-migration wound-healing assays, cells were plated into 6-well plates. After cells had grown to confluence, a wound was made across the well with a blue tip using the guide of a ruler. The gap of the wound was marked and imaged immediately after wounding. The gap was measured at different time points. Each experiment was performed in triplicate.

### *In vivo* study of tumor growth in SCID mice

SCID mice were obtained from the Center of Experimental Animals, Peking University Health Science Center. Five-week-old female mice were used. HIP-55 knockdown or HIP-55 overexpressing A549 cells were injected into subcutaneous sites on the shoulders of SCID mice. Six mice were used for each group. At seven weeks after inoculation, the mice were sacrificed and the tumors were weighed. The tumors were then homogenized and protein was extracted for Western blot with antibodies specific for HIP-55 or GAPDH.

### Statistical analysis

A student's t-test was used to compare individual data points among each group. The level of significance was defined as P<0.05.

## References

[1] Good MC, Zalatan JG, Lim WA (2011). Scaffold proteins: hubs for controlling the flow of cellular information. Science.

[2] Reinhardt HC, Yaffe MB (2013). Phospho-Ser/Thr-binding domains: navigating the cell cycle and DNA damage response. Nat Rev Mol Cell Biol.

[3] Lin CC, Melo FA, Ghosh R, Suen KM, Stagg LJ, Kirkpatrick J, Arold ST, Ahmed Z, Ladbury JE (2012). Inhibition of basal FGF receptor signaling by dimeric Grb2. Cell.

[4] Aitken A (2006). 14-3-3 proteins: a historic overview. Semin Cancer Biol.

[5] Chen S, Synowsky S, Tinti M, MacKintosh C (2011). The capture of phosphoproteins by 14-3-3 proteins mediates actions of insulin. Trends Endocrinol Metab.

[6] Fu H, Subramanian RR, Masters SC (2000). 14-3-3 proteins: structure, function, and regulation. Annu Rev Pharmacol Toxicol.

[7] Muslin AJ, Tanner JW, Allen PM, Shaw AS (1996). Interaction of 14-3-3 with signaling proteins is mediated by the recognition of phosphoserine. Cell.

[8] Yaffe MB, Rittinger K, Volinia S, Caron PR, Aitken A, Leffers H, Gamblin SJ, Smerdon SJ, Cantley LC (1997). The structural basis for 14-3-3:phosphopeptide binding specificity. Cell.

[9] Jin J, Smith FD, Stark C, Wells CD, Fawcett JP, Kulkarni S, Metalnikov P, O'Donnell P, Taylor P, Taylor L, Zougman A, Woodgett JR, Langeberg LK, Scott JD, Pawson T (2004). Proteomic, functional, and domain-based analysis of in vivo 14-3-3 binding proteins involved in cytoskeletal regulation and cellular organization. Curr Biol.

[10] Meek SE, Lane WS, Piwnica-Worms H (2004). Comprehensive proteomic analysis of interphase and mitotic 14-3-3-binding proteins. J Biol Chem.

[11] Pozuelo Rubio M, Geraghty KM, Wong BH, Wood NT, Campbell DG, Morrice N, Mackintosh C (2004). 14-3-3-affinity purification of over 200 human phosphoproteins reveals new links to regulation of cellular metabolism, proliferation and trafficking. Biochem J.

[12] Ensenat D, Yao Z, Wang XS, Kori R, Zhou G, Lee SC, Tan TH (1999). A novel src homology 3 domain-containing adaptor protein, HIP-55, that interacts with hematopoietic progenitor kinase 1. J Biol Chem.

[13] Kessels MM, Engqvist-Goldstein AE, Drubin DG (2000). Association of mouse actin-binding protein 1 (mAbp1/SH3P7), an Src kinase target, with dynamic regions of the cortical actin cytoskeleton in response to Rac1 activation. Mol Biol Cell.

[14] Larbolette O, Wollscheid B, Schweikert J, Nielsen PJ, Wienands J (1999). SH3P7 is a cytoskeleton adapter protein and is coupled to signal transduction from lymphocyte antigen receptors. Mol Cell Biol.

[15] Qi W, Liu X, Chen W, Li Q, Martinez JD (2007). Overexpression of 14-3-3gamma causes polyploidization in H322 lung cancer cells. Mol Carcinog.

[16] Zhang L, Wang H, Liu D, Liddington R, Fu H (1997). Raf-1 kinase and exoenzyme S interact with 14-3-3zeta through a common site involving lysine 49. J Biol Chem.

[17] Petosa C, Masters SC, Bankston LA, Pohl J, Wang B, Fu H, Liddington RC (1998). 14-3-3zeta binds a phosphorylated Raf peptide and an unphosphorylated peptide via its conserved amphipathic groove. J Biol Chem.

[18] Wang B, Yang H, Liu YC, Jelinek T, Zhang L, Ruoslahti E, Fu H (1999). Isolation of high-affinity peptide antagonists of 14-3-3 proteins by phage display. Biochemistry-Us.

[19] Datta SR, Dudek H, Tao X, Masters S, Fu H, Gotoh Y, Greenberg ME (1997). Akt phosphorylation of BAD couples survival signals to the cell-intrinsic death machinery. Cell.

[20] Wilson NS, Dixit V, Ashkenazi A (2009). Death receptor signal transducers: nodes of coordination in immune signaling networks. Nat Immunol.

[21] Hainaut P, Plymoth A (2013). Targeting the hallmarks of cancer: towards a rational approach to next-generation cancer therapy. Curr Opin Oncol.

[22] Li Z, Zhao J, Du Y, Park HR, Sun SY, Bernal-Mizrachi L, Aitken A, Khuri FR, Fu H (2008). Down-regulation of 14-3-3zeta suppresses anchorage-independent growth of lung cancer cells through anoikis activation. Proc Natl Acad Sci U S A.

[23] Han J, Kori R, Shui JW, Chen YR, Yao Z, Tan TH (2003). The SH3 domain-containing adaptor HIP-55 mediates c-Jun N-terminal kinase activation in T cell receptor signaling. J Biol Chem.

[24] Hu MC, Qiu WR, Wang X, Meyer CF, Tan TH (1996). Human HPK1, a novel human hematopoietic progenitor kinase that activates the JNK/SAPK kinase cascade. Genes Dev.

[25] Kiefer F, Tibbles LA, Anafi M, Janssen A, Zanke BW, Lassam N, Pawson T, Woodgett JR, Iscove NN (1996). HPK1, a hematopoietic protein kinase activating the SAPK/JNK pathway. Embo J.

[26] Kessels MM, Engqvist-Goldstein AE, Drubin DG, Qualmann B (2001). Mammalian Abp1, a signal-responsive F-actin-binding protein, links the actin cytoskeleton to endocytosis via the GTPase dynamin. J Cell Biol.

[27] Johnson C, Tinti M, Wood NT, Campbell DG, Toth R, Dubois F, Geraghty KM, Wong BH, Brown LJ, Tyler J, Gernez A, Chen S, Synowsky S, MacKintosh C (2011). Visualization and biochemical analyses of the emerging mammalian 14-3-3-phosphoproteome. Mol Cell Proteomics.

[28] Ramalingam SS, Owonikoko TK, Khuri FR (2011). Lung cancer: New biological insights and recent therapeutic advances. CA Cancer J Clin.

[29] Han J, Shui JW, Zhang X, Zheng B, Han S, Tan TH (2005). HIP-55 is important for T-cell proliferation, cytokine production, and immune responses. Mol Cell Biol.

[30] Bae SM, Min HJ, Ding GH, Kwak SY, Cho YL, Nam KH, Park CH, Kim YW, Kim CK, Han BD, Lee YJ, Kim do K, Ahn WS (2006). Protein expression profile using two-dimensional gel analysis in squamous cervical cancer patients. Cancer Res Treat.

[31] Kim HN, Choi DW, Lee KT, Lee JK, Heo JS, Choi SH, Paik SW, Rhee JC, Lowe AW (2007). Gene expression profiling in lymph node-positive and lymph node-negative pancreatic cancer. Pancreas.

[32] Liu N, Sun N, Gao X, Li Z (2014). Phosphosite Mapping of HIP-55 Protein in Mammalian Int J Mol Sci.

[33] Li Z, He K, Pan C, Liu N (2013). Mass Spectrometric Analysis of Phosphorylation Modification in 14-3-3 Protein. Chinese J Anal Chem.

[34] Wang H, Song X, Logsdon C, Zhou G, Evans DB, Abbruzzese JL, Hamilton SR, Tan TH, Wang H (2009). Proteasome-mediated degradation and functions of hematopoietic progenitor kinase 1 in pancreatic cancer. Cancer Res.

[35] Vogelstein B, Papadopoulos N, Velculescu VE, Zhou S, Diaz LJ, Kinzler KW (2013). Cancer genome landscapes. Science.

[36] Boateng LR, Cortesio CL, Huttenlocher A (2012). Src-mediated phosphorylation of mammalian Abp1 (DBNL) regulates podosome rosette formation in transformed fibroblasts. J Cell Sci.

[37] Le Bras S, Moon C, Foucault I, Breittmayer JP, Deckert M (2007). Abl-SH3 binding protein 2, 3BP2, interacts with CIN85 and HIP-55. Febs Lett.

[38] Mise-Omata S, Montagne B, Deckert M, Wienands J, Acuto O (2003). Mammalian actin binding protein 1 is essential for endocytosis but not lamellipodia formation: functional analysis by RNA interference. Biochem Biophys Res Commun.

[39] Schymeinsky J, Sperandio M, Walzog B (2011). The mammalian actin-binding protein 1 (mAbp1): a novel molecular player in leukocyte biology. Trends Cell Biol.

[40] Xu W, Stamnes M (2006). The actin-depolymerizing factor homology and charged/helical domains of drebrin and mAbp1 direct membrane binding and localization via distinct interactions with actin. J Biol Chem.

[41] Masters SC, Fu H (2001). 14-3-3 proteins mediate an essential anti-apoptotic signal. J Biol Chem.

[4] Goldman EH, Chen L, Fu H (2004). Activation of apoptosis signal-regulating kinase 1 by reactive oxygen species through dephosphorylation at serine 967 and 14-3-3 dissociation. J Biol Chem.

[4] Puckett MC, Goldman EH, Cockrell LM, Huang B, Kasinski AL, Du Y, Wang CY, Lin A, Ichijo H, Khuri F, Fu H (2013). Integration of apoptosis signal-regulating kinase 1-mediated stress signaling with the Akt/protein kinase B-IkappaB kinase cascade. Mol Cell Biol.

